# Myocardium tissue changes caused by electrical transthoracic discharges in rats

**DOI:** 10.1186/1755-7682-2-31

**Published:** 2009-10-23

**Authors:** Marcelo Ferreira, Celso Ferreira, Luiz Carlos de Abreu, Vitor E Valenti, Neif Murad, Adriano Meneghini, Celso F Filho, Japy Angelini de Oliveira Filho

**Affiliations:** 1 Department of Clinical Medicine, Cardiology Division, School of Medicine of ABC, Santo André, SP, Brazil; 2 Department of Morphology and Physiology, School of Medicine of ABC, Santo André, SP, Brazil; 3 Department of Medicine, Federal University of São Paulo (Unifesp), São Paulo, SP, Brazil

## Abstract

**Background:**

Cardiomyocytes cytoarchitecture changes caused by transthoracic countershocks have been focused recently. We aimed to evaluate the effects of electrical discharge application in the mitochondria structure in atrial myocardium of rats.

**Methods:**

An electrical cardioverter was adapted to small rodent animals for our research. Electrical discharges were applied to the precordial region of 30 albino rats: (1) control group - animals that remained on resting period and were afterwards sacrificed; (2) electrical discharge group - animals that remained on resting period, followed by ten electrical discharges of 300 mV and sacrificed, and; (3) electrical post-discharge group - animals that remained on a resting period and received ten electrical discharges like the electrical discharge group, but were sacrificed seven days subsequently. We examined liver, adrenal and left atrium tissue fragments of the three groups.

**Results:**

It was observed in control and post-discharge groups a normal cellular structure aspect with preserved architecture of cardiomyocytes and continuous sarcoplasmic membrane integrity. On the other hand, cardiac muscle fibers with mitochondrial edema and lysis occurred in the discharge group. Glycogen and adrenal lipids were not depleted in all groups.

**Conclusion:**

These data suggest that transthoracic electrical discharges induce mitochondrial injuries in atrial cardiac cells of rats.

## Introduction

Previous studies have already demonstred cardiomyocytes injuries caused by electrical discharges. Generally after defibrillation, clinical experience discloses that an electrical energy pulse applied has sufficient energy to preserve the heart effectively, even though discharge rises proportionally to patient body weight [1-4]. Moreover, the magnitude of damage depends on electrical pulse waves, so that myocardial injury is pointed out considering the unevenness of ST on the electrocardiogram and it was reported a minor aggression when truncated biphasic shocks were applied instead of attenuated monophasic sinusoidal discharges [5]. Different clinical manifestations occur after electrical discharges. It was observed destroyed nuclei, extensive myofibrillar contraction and harmed intercalated disks in atrial subcellular mechanisms. Critical analyses allow these manifestations to be attributed to underlying heart diseases, pre-existing metabolic deviations, or to cardiorespiratory arrests that caused them, and not selectively as a consequence of this procedure [6,7]. Concerning the instantaneous myocardial degeneration after cardiorespiratory post-failure phase for 10 minutes, the cardiovascular recovery probability is practically null [8].

Ultrastructural analysis of mitochondria is very important, and as an organelle which is extremely sensitive to hypoxia, it represents a structure of central importance in aerobic metabolism. Mitochondria are the powerhouses of the cell where oxidative phosphorylation takes place to generate ATP. Oxidative phosphorylation involves the transfer of electrons to oxygen coupled to the synthesis of ATP. Oxidative stress is the increased generation of free radicals resulting in oxidative damage to DNA, proteins, and lipids. Impairment of mitochondrial oxidative phosphorylation is associated with increased oxidative stress [9].

Although different clinical manifestations occur after electrical discharges [6,7], no study evaluated mitochondrial profile in cardiomyocytes immediately after electrical discharges. Therefore, we enderavored to evaluate mitochondrial cristolysis in atrial myocardium tissue of rats exposed to successive transthoracic electrical discharges.

## Method

### Animals

All procedures were performed in accordance with ethical guidelines of the National Institutes of Health Guide for the Care and Use of Laboratory Animals and were approved by the Ethical Committee in research of our University. Experiments were performed on thirty adult male rats (Rattus *novergicus albinus*, Rodentia Mammalia), weighing 250-340 grams. Temperature was 22°C, air humidity nearly 60% and the clear-dark cycle was controlled and established with twelve hours each one. After an adaptation period of nearly one week, animals were randomly selected and separated into three groups: Control Group (C, n = 10): animals that received food and water ad libitum and were kept at room temperature for seven consecutive days; Electrical discharge group (D, n = 10): Animals remained on resting period with water and food ad libitum, followed by ten 300 mV electrical discharges and; Electrical post-discharge group (PD, n = 10): Animals received ten electrical discharges (300 mV) and were fed with water and food ad libitum for seven consecutive days.

### Transthoracic electrical discharges

Electrodes adapted for this study (3 M trademark) were positioned on animals' precordial region in order to apply the electrical discharges. The handling of the electrical discharges was through an electric cardioverter equipment adapted to small rodent animals by the Bioengineering department of our University. The equipment was assembled on the basis of the same used for cardiac rhythm reversion disorders in human beings. The cardioverter device has a potential to generate serial current impulses of a maximal 300 mV (three joules equable). This serial current with combined signal pattern depicts a capacitor discharge in a RL circuit (coil and resistance), within 10 ms average. The impedance of the developed system for the experimental purpose was not determined, based on the complexity and nature in these kinds of study limitations. However, experimental group conditions were always equable, so that comparison among them could be reliable.

### Histological Procedures

Immediat ely after light ether anesthesia, we verified tail tonus and response to external stimuli before and during surgical procedures through evaluation of vibrissa movements, all animals were submitted to a thoracotomy. The thorax of each rat was opened and the left atrium exposed and removed. Cold 2% glutaraldehyde solution was poured on the heart still beating in order to prevent other ultra structural modifications. Left atrial myocardium fragments were fixed in 3% formaldehyde with 5% glutaraldehyde in a 2% phosphate buffer solution (KH_2_PO_4_) at 4°C (0.1 M; pH = 7.4). Fragments were cut into small pieces of 1 mm^3 ^and post-fixed in a 1% OsO_4 _solution for 2 hours, dehydrated and embedded in araldite. Silver or gray thin sections (60-90 nm) were selected on a Porter-Blum MT-B ultramicrotome. The ultra-slices were mounted on copper silver grids with 200 patches and stained with uranyl acetate and lead citrate. In addition, based in previous studies [9,10], in order to evaluate stress feature, two pieces of left liver lobe and right adrenal gland were removed by laparotomy technique for light microscopy investigation.

### Data analysis

The mitochondrial morphology was analyzed under electron microscopy, with emphasis on the cristae integrity. Mitochondrial lesions were defined as a partial or complete cristalysis and their substitution by lacunar areas. For each group of randomly examined animals, nine electron micrographs of the left atrial wall were obtained. The samples were examined by two independent investigators with the same and standardized criteria [9,10]. Counting was done in order to determine how many injured mitochondria were presented in each sample analysis, as well as the means of injured mitochondria (Table 1). The number of injured mitochondria compared to the amount was entitled "crystolysis index" [9,10].

**Table 1:** Groups with intact and with crystolysis mitochondrial cells; number and percentage for the three experimental rats groups.

***Mitochondria***	***C***	***D***	***PD***
***Crystolysis***	26	596	309
***Intact***	602	97	203
***Total***	625	694	510
***Indexes***	3.8*	84.75*	60.84*

*Differ significantly (p < 0.005); C - Control group; D-Electrical discharge group; PD - Electrical post-discharge group.

### Statistical Analysis

In order to evaluate the data associated to the crystolysis index, in different groups, chi-square and chi-square partition tests were used (Table 2). In order to evaluate the data associated to crystolysis index, comparison among independent groups, Kruskall-Wallis and Tukey post-hoc tests were applied. Concordance of measurements performed by the two investigators was evaluated and analyzed by *Bartko's *intra-class correlation coefficient according to Fleiss [11] guidelines:

**Table 2:** Chi-square test.

X^2^-Critical = 5.83*	X^2^-Determined = 925*
***Chi-square partition test***	
***C X D***	Determined X^2 ^= 925**
***C X PD***	Determined X^2 ^= 437**
***D X PD***	Determined X^2 ^= 95.9**

*Differ significantly (P < 0.00001); **Differs significantly (P < 0.0001).

### Bartko's test formula



**R**: Bartko's correlation index; **PMS**: Patients Mean Square; **RMS**: Researcher Mean Square; **EMS**: Error Mean Square; **N**: Number of events; **K**: Number of investigators

The level of significance was set at p < 0.05 in all analyses.

## Results

**Figure 1:**
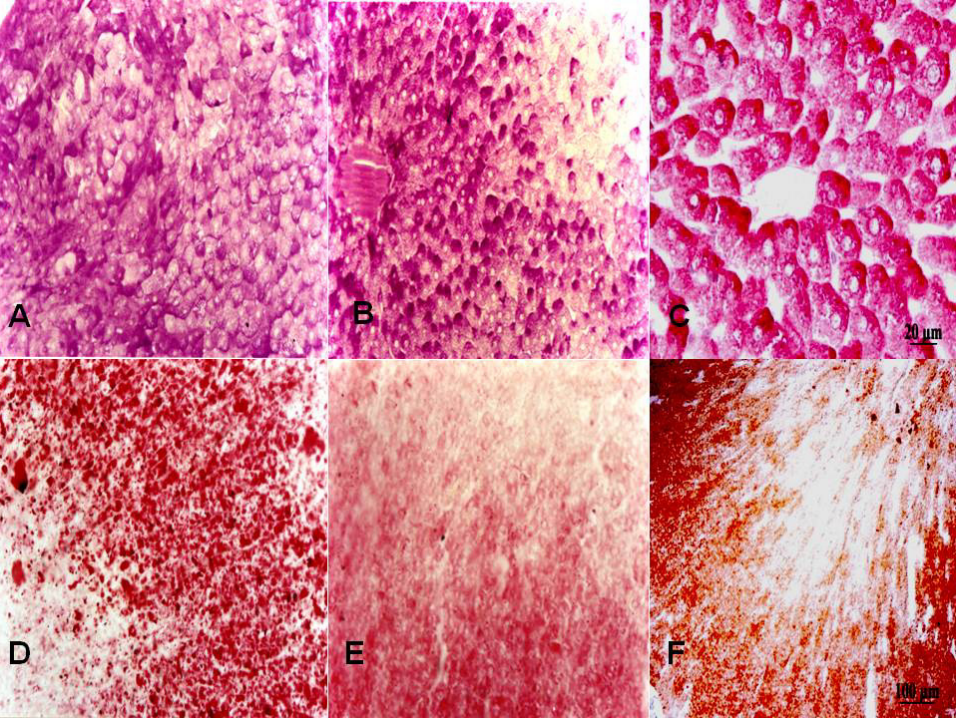
**Glycogen (blushed with PAS method) and lipid (blushed with Sudan IV) depletion in hepatocytes and adrenal gland cells, respectively, were verified to examine stress condition immediately after countershock**. There was no significative difference among control (A-D), post-discharge (B-E) and discharge (C-F) groups regarding glycogen depletion in hepatocytes (A, B and C) and lipid depletion in adrenal gland cells (D, E and F).

In adrenal and liver tissue samples it was observed no alteration on lipid and glycogen depletion among the three groups, respectively (Figure 1).

To clarify whether transthoracic electrical discharges promote cardiomyocytes injuries, we performed a morphological evaluation study in different experimental groups. Comparisons among control (C), Electrical discharge (D) and Electrical post-discharge (PD) were performed (Table 3 and Table 4).

**Table 3:** Comparative Analysis of Mitochondrial Crystalysis Profile Number.

***Numbers***	***C***	***D***	***PD***
***1***	3.9	87.5	15.0
***2***	3.0	61.0	66.0
***3***	7.0	85.5	32.0
***4***	2.0	75.0	55.0
***5***	2.5	53.0	75.0
***6***	1.5	3.0	43.0
***7***	2.0	44.5	28.0
***8***	2.0	84.0	42.0
***9***	4.0	102.5	44.5
***Mean***	3.1	66.22	44.5

**Table 4:** Values of Mitochondrial Profiles with Crystalysis for Nine Photomicrographs Analysed by Two Independent Observers (O1 and O2) in the Control (C), Electrical discharge (D) and Electrical post-discharge (PD) Groups.

***Photo***	***C***		***D***		***PD***	
	O1	O2	O1	O2	O1	O2
***1***	4	3	92	84	20	16
***2***	4	2	72	53	58	72
***3***	6	4	90	85	29	31
***4***	0	3	75	66	58	52
***5***	2	2	2	51	76	72
***6***	1	4	4	0	53	29
***7***	2	2	48	42	39	19
***8***	0	3	85	80	44	42
***9***	5	5	102	102	45	46

***Total***	2.67	3.11	63.33	62.55	46.88	42.11

The values represented in the columns express the mean result between two independent observers in the control (C), discharge (D) and post-discharge (PD). Variance analysis was performed using the Kruskal-Wallis method. Hc = 5.79*, Hcalc. = 16.47*(*P < 0.001). Comparison of means> Zc. = 2.6 and Zcalc. = 10.77**(**P < 0.005). D > PD > C.

**Figure 2:**
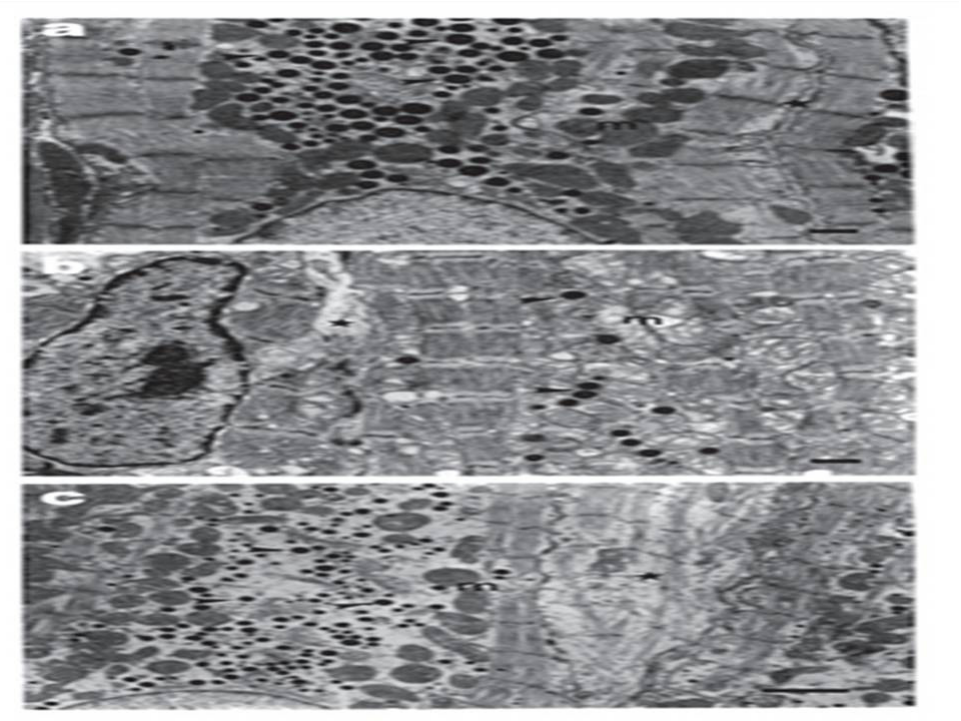
**Electromicrographs of left atrial cardiomyocytes sliced longitudinally**. In (a): control group - sarcomere clearly delimited by the z lines (star), normal aspect of mitochondria (m) and paranucleate granules (arrows) with electron dark appearance were observed. In (b): electrical discharge group - note the high number of dropsy damaged mitochondria, disarranged sarcomere (star) and dispersed electrondense granules presence (arrows). In (c): post-discharge group - preserved mitochondria and electrondense granules dispersion tendency at the paranucleate region were verified seven days later to electrical discharges. Tangential cardiomyocyte image is visible (star). Amplification × 13.200.

Our electron microscopy analysis presented in D and PD (Figure 2) groups showed regions with normal aspect and intermyofibrillar areas. Those regions were fulfilled with fibrous and amorphous conjunctive cellular tissue. Intercellular spaces were apparently amplified in other areas. Instead, greatly injured cells or cells with well conserved subcellular components in different areas were discovered. In C group, (Figure 2) we observed mitochondrial common aspect, paranuclear granules with electron dense manifestation and sarcomere clearly enclosed by the z lines.

It was verified in D group (Figure 2) cardiomyocytes with very intense alterations inside the mitochondria. There was lysis, erasing and edema in different degrees, reaching substitution disappearance of the cristae, giving place to lacunar areas (crystolysis). Myofibrils occasionally showed a heterogeneous pattern with discontinuous appearance, although the structural aspect was similar to the normal shape. Some myofilament areas were displaced, disintegrated and lacking sarcoplasmic elements. Around the contraction bands, some myofilament zones disappeared and were replaced by a limited necrosis mechanism with homogenous characteristic

In the PD group (Figure 2) we observed intact cardiomyocytes. The sarcoplasmic membrane, in bulk areas, had a continuous and rectilinear appearance. The sarcoplasm full of mitochondria of different shapes and dimensions was interposed between myofilaments. Myofilaments were normally arranged with some regions of discontinuity and the nucleus, generally single and central, showed an euchromatic archetype. In our morphometric analysis, two independent investigators measured number and means of mitochondrial counting profile with crystolysis. Our findings presented in Tables 1 and 2 indicate that the high index of intraclass correlation (R) ranged between 0.60 to 0.99 values, which validate the reliability of this method for assessing differences among the groups (Table 2). For each animal studied, the means between both investigators were considered, bearing in mind the reproducibility among them. Statistically significant differences among the groups were demonstrated and they were complemented by a test of difference among the means. D group was statistically different compared to C and PD groups. PD group showed intermediate values between C and D groups.

## Discussion

Restricted papers regarding this method of analysis led us to recommend a morphometrical study of changes in atrial cardiomyocytes caused by electrical discharges. An intense cellular disorder was observed in D group, particularly in mitochondria, which presented destroyed cristae and edema. The significantly difference between D and PD groups suggests that myocardial subcellular components tend to recover in a given period of time. Despite the elevated number of injured mitochondria, neither fatality nor health-state compromising trace was verified at the successive period to the discharges in PD group. A strong hypothesis relating other compensatory mechanisms to physiologically unviable mitochondria in the cardiac cells must also be considered. Mitochondria damage and supposed ATP reduced production justifies myocardial evidenced dysfunction at post cardioversion and defibrillation conditions [12].

In D group, we observed high intensity changes in mitochondria inside the cardiac muscle fibers. It is worth to pointing out that mitochondrial cells are involved in alternative roles and not only as a single source of energy production, but also in intracellular calcium homeostasis modulation, apoptosis and thermogenesis regulation [13,14]. Cellular ATP depletion happens due to mitochondrial disorder conditions, which led to cellular loss by necrosis. Intracellular calcium homeostasis effects were likely occurred by blood reperfusion and ischemia. Nevertheless, cellular death does not always happen through necrosis, but alternatively by apoptosis. In this situation, the interference of two proteins located at mitochondrial inter-membrane space, cytochrome C and apoptosis induction factor, migrate to the cytoplasm and trigger the apoptosis process [14].

The analysis of myocardial consequence on electric cardioversion and defibrillation situations (employing either molecular biology techniques 3 or indirect methods like electrocardiogram 5), expresses varied clinical outstanding viewpoints. Following a serious analysis considering underlying heart disorders, the pre-existence of metabolic defects or those arising from cardiorespiratory dysfunctions [15], electrical discharge application is normally chosen, and it is not exclusively selective compared to other types of clinical procedures.

PD group was planned to evaluate the late systemic and myocardial involvement caused by adrenergic release. Immediately after the electrical discharges or on the seventh day subsequent to these experimental procedures we did not observe hepatic glycogen and adrenal lipid depletion in any preparation. Because of the animal adaptation period during the rest stage, the chance of dehydration, malnutrition, acid-base balance bearers and metabolic disorders were eliminated. In addition, due to the short time that elapsed between the discharges and collection of material for verification, the systemic interference of catecholamines liberation is hesitant.

Electrical discharges in chicken cell culture in vitro were studied and new evidences showed that temporary microlesions were produced in cellular sarcolemma [16]. Other experiments in dogs confirmed the effects of direct current discharges on mitochondria. Many mitochondrial pathological aspects like loss of membrane integrity, swollen units and disruption were found even after application of endocardial low energy countershocks [4,12,17]. Considering the changes shown by electron microscopy at the seventh day in our procedures, two possibilities should be considered: constant effects based on catecholaminergic action and/or the remission changes derived from electrical discharges were caused by electrical discharge stress condition. In both cases, adrenal and liver tissues did not show lipid or glycogen depletion, respectively, in that the space of time was sufficiently distant from the moment in which the electrical discharges were applied. Late morphological effects related to countershocks were investigated in order to consider the possibility of systemic and atrial modifications. Alterations interfere functionally in clinical complications, although at these situations exclusively electric current flow effects were not intended to be discriminate. It is important to declare that either from catecholaminergic or electrical discharge injury, effects must be evaluated.

Our study is helpful, since important information should be considered in clinical application field for myocardial protection, before and after clinical proceedings associated to transthoracic electrical discharges, particularly in subjects with heart disorders. However, our study has some limitations, we did not examine systolic and diastolic blood pressure, heart rate, contractility, calcium homeostasis, TnI levels as well as others injury indicators, which could complement the countershock effects on heart and cardiovascular parameters. We only showed electrical discharges effects on mitochondria of atrial cardiomyocytes.

In summary, our data indicate that countershock induces mitochondrial crystolysis increase in atrial cardiac cells.

## Competing interests

The authors declare that they have no competing interests.

## Authors' contributions

MF, CFF and CF performed the experimental procedures and helped to write the manuscript. VEV, NM, AM and LCA carried out the statistical analysis and participated in design the manuscript and all authors read and approved the final manuscript.

## References

[B1] Ehsani A, Ewy GA, Sobel BE (1976). Effects of electrical countershocks on serum creatine phosphokinase (CPK) isoenzyme activity. Am J Cardiol.

[B2] Babbs CF, Tacker WA, VanVleet JF, Bourland JD, Geddes LA (1980). Therapeutic indices for transchest defibrillator shocks: effective, damaging, and lethal electrical doses. Am Heart J.

[B3] Jones JL, Jones RE, Balasky G (1987). Microlesion formation in myocardial cells by high intensity electric field stimulation. Am J Physiol.

[B4] Dhoble A, Puttarajappa C, Neiberg A (2008). Dermatomyositis and supraventricular tachycardia. Int Arch Med.

[B5] Bardy GH, Marchlinski FE, Sharma AD, Worley SJ, Luceri RM, Yee R, Halperin BD, Fellows CL, Ahern TS, Chilson DA, Packer DL, Wilber DJ, Mattioni TA, Reddy R, Kronmal RA, Lazzara R (1996). For the transthoracic investigator - Multicenter comparison of truncated biphasic shocks and standard damped sine wave monophasic shocks for transthoracic ventricular defibrillation. Circulation.

[B6] American Heart Association (1992). Guidelines for cardiopulmonary resuscitation and emergency cardiac care. JAMA.

[B7] (2005). Proceedings of the 2005 International Consensus on Cardiopulmonary Resuscitation and Emergency Cardiovascular care Science with Treatment Recommendations. Resuscitation.

[B8] Cummins RO (1989). From concept to standard-of-care? Review of the clinical experience with automated external defibrillators. Ann Emerg Med.

[B9] Meneghini A, Ferreira C, Abreu LC, Ferreira M, Ferreira Filho C, Valenti VE, Murad N (2008). Cold stress effects on cardiomyocytes nuclear size in rats: light microscopic evaluation. Rev Bras Cir Cardiovasc.

[B10] Daud FV, Murad N, Meneghini A, Ferreira M, Filho CF, Abreu LC, Valenti VE, Ferreira C (2009). Fluoxetine effects on mitochondrial ultrastructure of right ventricle in rats exposed to cold stress. Rev Bras Cir Cardiovasc.

[B11] Fleiss JL (1989). A critique of recent research on the two-treatment crossover design. Control Clin Trials.

[B12] Schirmer U, Hemmer W, Lindner KH, Anhäupl T, Wieser T (1997). Ultrastructural alterations in the right and left ventricular myocardium following multiple low energy endocardial countershocks in anesthetized dogs. Pacing Clin Electrophysiol.

[B13] Hassouna A, Loubani M, Matata BM, Fowler A, Standen NB, Galiñanes M (2006). Mitochondrial dysfunction as the cause of the failure to precondition the diabetic human myocardium. Cardiovasc Res.

[B14] Kowaltowski AJ (2000). Alternative mitochondrial functions in cell physiopathology: beyond ATP production. Braz J Med Biol Res.

[B15] Moller DE, Kaufman KD (2005). Metabolic syndrome: perspective. Annu Rev Med.

[B16] Jones DL, Narayanan N (1998). Defibrillation depresses hear sarcoplasmic reticulum calcium pump: a mechanism of postshock dysfunction. Am J Physiol Heart Circ Physiol.

[B17] Scotland RS, Ahluwalia A, Hobbs AJ (2005). C-type natriuretic peptide in vascular physiology and disease. Pharmacol Ther.

